# Chiral gels derived from secondary ammonium salts of (1*R*,3*S*)-(+)-camphoric acid

**DOI:** 10.3762/bjoc.6.100

**Published:** 2010-09-21

**Authors:** Tapas Kumar Adalder, N N Adarsh, Ravish Sankolli, Parthasarathi Dastidar

**Affiliations:** 1 Indian Association for the Cultivation of Science, Kolkata, India; 2 Indian Institute of Science, Bangalore, India

**Keywords:** crystal engineering, LMOG, single crystal X-ray diffraction, supramolecular gels, supramolecular synthon

## Abstract

In order to have access to chiral gels, a series of salts derived from (1*R*,3*S*)-(+)-camphoric acid and various secondary amines were prepared based on supramolecular synthon rationale. Out of seven salts prepared, two showed moderate gelation abilities. The gels were characterized by differential scanning calorimetry, table top rheology, scanning electron microscopy, single crystal and powder X-ray diffraction. Structure property correlation based on X-ray diffraction techniques remain inconclusive indicating that some of the integrated part associated with the gelation phenomena requires a better understanding.

## Introduction

A gel is a two component system which is mainly liquid with a very little amount of solid. In gel state, gelator molecules form 3-D networks within which solvent molecules are trapped thus resulting in a gel. Depending on the nature of the network, gels can be of two kinds – chemical or polymeric and physical or supramolecular. While covalent bonds are responsible for the formation of 3-D networks in chemical gels, various non-covalent interactions such as hydrogen bonding, π-π stacking, hydrophobic, van der Waals forces etc. are required to form gel network in supramolecular gels. It is believed that in supramolecular gels, the gelator molecules self-assemble to form self-assembled fibrilar networks (SAFINs) which, by some means, are entangled to form 3-D gel networks within which the solvent molecules are immobilized via capillary force action to form gel. A gel with an organic solvent is called organogel whereas that obtained from water or an aqueous solvent mixture is known as a hydrogel. Among the various classes of supramolecular gelators, interest in low molecular mass organic gelators (LMOGs) [1–10] is a continuous expanding area on account of their various promising applications [11–13]. Broadly, LMOGs are used in cosmetics [14], tissue engineering [15], drug delivery and biomedical applications [16–19], art conservation [20–22], templated synthesis of nanoparticles [23–24], capture and removal of pollutants [25], catalysis [26], sensors [27], electrooptics/photonics [28], structure-directing agents [29–30] etc. The gelator molecules form SAFINs typically when a hot solution containing a small amount of gelator is cooled below a critical temperature (sol-gel temperature); the SAFINs then start to entangle themselves to form a three dimensional network within which the solvent molecules are immobilized by capillary force interactions resulting in gel formation. The elegance of a LMOG lies in the reversible nature of the gel forming network and it is possible to tune the physical properties of the gel by applying external stimuli such as temperature, pH, sound waves [31], anions [32] etc.

**Scheme 1:**
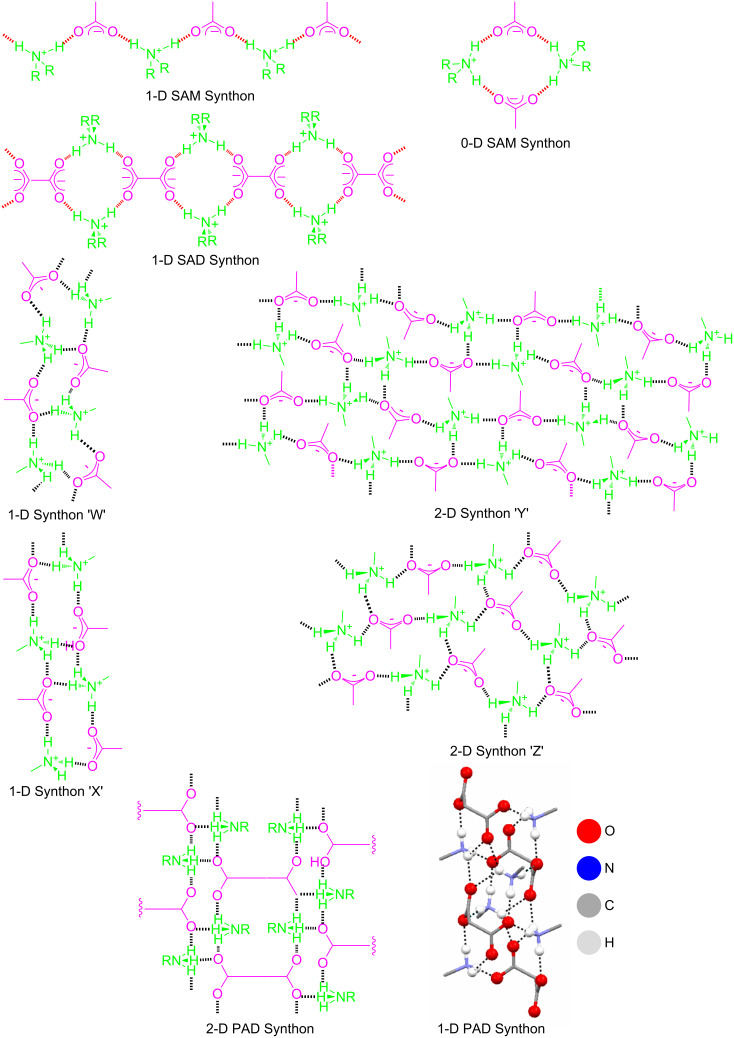
Different types of 1-D and 2-D HBN forming supramolecular synthons.

The lack of understanding of the mechanism of gel formation at the molecular level makes it difficult to design a gelator. Most of gelling agents have been discovered serendipitously or derived from a known gelator scaffold. But recent advances in the supramolecular chemistry [33] and crystal engineering [34] has made it possible to design a gelator molecule in a rational manner by exploiting a supramolecular synthon [35] approach, at least for certain classes of gelling agents [3]. We have shown by correlating many single crystal structures of organic salts derived from various organic acids (both mono- and di-basic) and amines (both primary and secondary) with their gelling and non-gelling behavior that 1-D and 2-D forming supramolecular synthons such as secondary ammonium monocarboxylate (SAM) [36–37], secondary ammonium dicarboxylate (SAD) [38–39], primary ammonium monocarboxylate (PAM) [40–41] and primary ammonium dicarboxylate (PAD) [42–43] appear to play a crucial role in gel formation (Scheme 1).

**Scheme 2:**
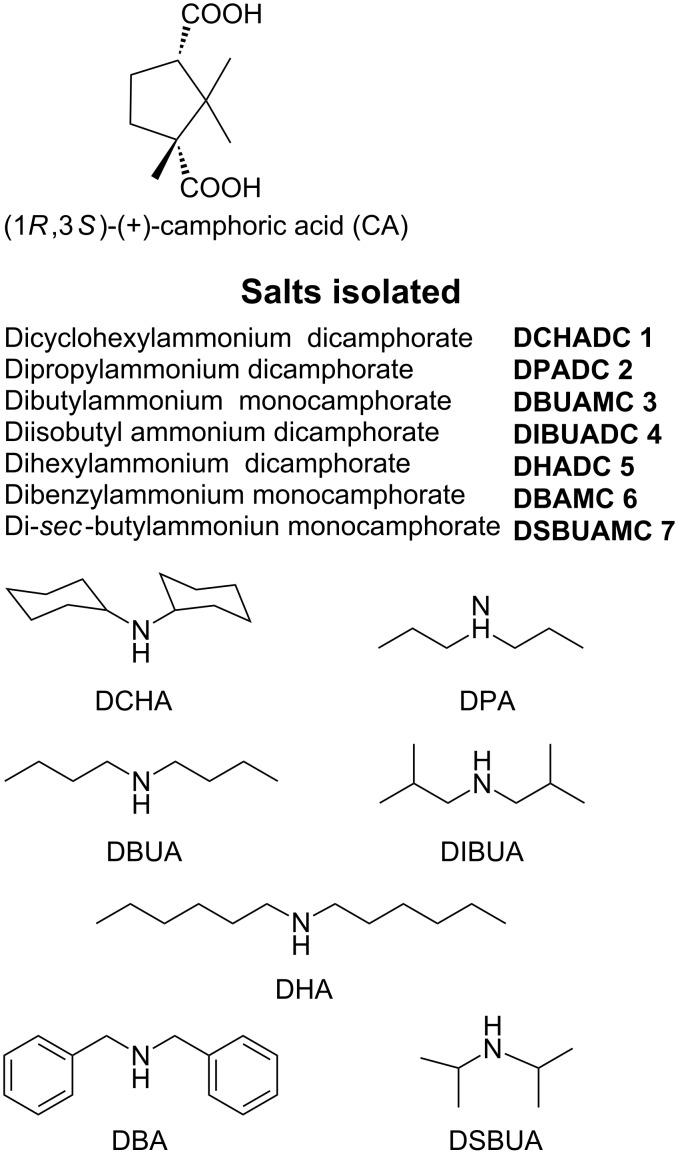
Salts studied in the present report.

In the present work we intend to exploit **SAD** synthons to make chiral gels. Supramolecular chirality is an important aspect in the development of chiral catalysts [26], chiro-optical switches [44], helical crystallization of proteins and inorganic replicas [45], chiral resolution [46] etc. For this purpose, we have reacted a dibasic acid such as (1*R*,3*S*)-(+)-camphoric acid with various secondary amines namely, dicyclohexylamine (DCHA), dipropylamine (DPA), dibutylamine (DBUA), diisobutylamine (DIBUA), dihexylamine (DHA), dibenzylamine (DBA) and di-*sec*-butylamine (DSBUA) in a 1:2 molar ratio (Scheme 2).

These salts were then used in gelation studies and the resulting gels characterized by table top rheology, differential scanning calorimetry (DSC), scanning electron microscopy (SEM), single- and powder X-ray diffraction (SXRD and PXRD, respectively). Single crystal structures of two gelators and one nongelator, i.e., DBUAMC **3,** DBAMC **6,** and DCHADC **1**, respectively were determined and discussed in the context of structure-property correlation.

## Results and Discussions

### Synthesis

The salts were isolated as crystalline solids by the slow evaporation of a methanolic solution of the acid and the corresponding amine taken in an appropriate molar ratio. FT-IR spectra indicated that both the protons of the dicarboxylic acids were absent as was evident from the presence of the characteristic band of COO^−^ (1622–1635 cm^−1^) and absence of COOH (1699 cm^−1^) in salts **1**, **2**, **4** and **5**. However, the presence of FT-IR bands at 1701, 1631 cm^−1^ for salt **3**, 1705, 1548 cm^−1^ for salt **6** and 1701, 1620 cm^−1^ for salt **7** clearly indicated that 1:1 acid:amine salts were formed in these cases; satisfactory elemental analysis also support the formation of 1:1 salts **6** and **7** when the corresponding acid and the amines were deliberately reacted in a 1:1 molar ratio. However, that was not the case with salt **3** whose elemental analysis data did not match a 1:1 stoichiometry (see Experimental).

#### Gelation Studies

All the salts were scanned for gelation in various solvents. In a typical procedure, 20 mg of a salt was taken in a test tube (10 mm × 100 mm) and dissolved in 0.5 ml of the solvent of choice by heating on a hot plate. The gel was obtained by keeping the solution undisturbed under ambient conditions (Table 1).

**Table 1:** Gelation data (CS = Clear solution, GP = Gelatinous precipitate, FC = Fibrous crystal, CP = crystalline precipitate, AP = Amorphous precipitate, WP = White precipitate, YP = Yellow precipitate, PG = Partial gel, WG = Weak gel, FGN = Fibrous gelatinous network, PLC = plate like crystal, WT = White turbidity).

Solvent	DCHDC 1	DPADC 2	DBUAMC 3	DIBUADC 4	DHADC 5	DBAMC 6	DSBUAMC 7

	MGC/Wt %	MGC/Wt %	MGC/Wt % (*T*_gel_/°C)	MGC/Wt %	MGC/Wt %	MGC/Wt % (*T*_gel_/°C)	MGC/Wt %
Bromobenzene	CS	FC	PG	FGP	WT	4.00 (98)	FC
Chlorobenzene	CS	WP	PG	FGP	WT	4.00 (110)	FC
1,2-Dichloro-benzene	CS	FC	PG	FC	WT	2.22 (106)	FC
Toluene	GP	CS	CS	CS	CS	AP	CS
*o*-Xylene	CS	CS	CS	CS	CS	AP	CS
*m*-Xylene	CS	CS	GP	CS	CS	CP	CS
*p*-Xylene	CS	CS	GP	CS	CS	CP	CS
Mesitylene	CS	CS	GP	CS	CS	WP	CS
Nitrobenzene	GP	YP	4.00 (78)	PLC	WG	YP	FC
1,4-Dioxane	FC	WP	FGN	FC	WG	AP	CS
Methylsalicylate	CS	CS	WG	PLC	CS	WP	SC
DMSO	FC	CS	FC	CS	FC	CS	CS
DMF	CP	CS	FC	CS	FC	CS	CS
EG	CS	CS	CS	CS	CS	FC	CS

**Figure 1:**
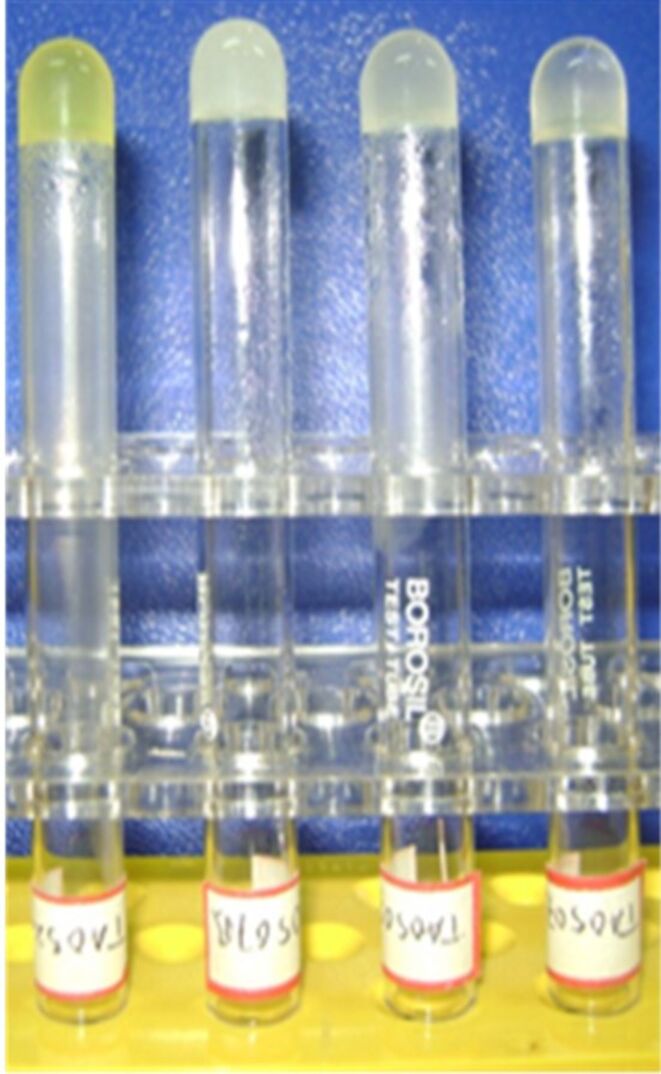
Photomicrographs of the organogels (from left to right: nitrobenzene gel of DBUAMC **3**; 1,2-dichlorobenzene gel of DBAMC **6**; chlorobenzene gel of DBAMC **6**; bromobenzene gel of DBAMC **6**).

The salts DBUAMC **3** and DBAMC **6** gave stable gels with polar solvents such as nitrobenzene, and bromobenzene, chlorobenzene and 1,2-dichlorobenzene, respectively. The salt DBUAMC **3** also gave a partial gel (PG) with bromobenzene, chlorobenzene, 1,2-dichlorobenzene; a gel is called PG when the top layer of the solution becomes gel-like entrapping the flowing liquid underneath [47]. DHADC **5** gave a weak gel with nitrobenzene and 1,4 dioxane. Representative photomicrographs of the organogels are depicted in Figure 1.

**Figure 2:**
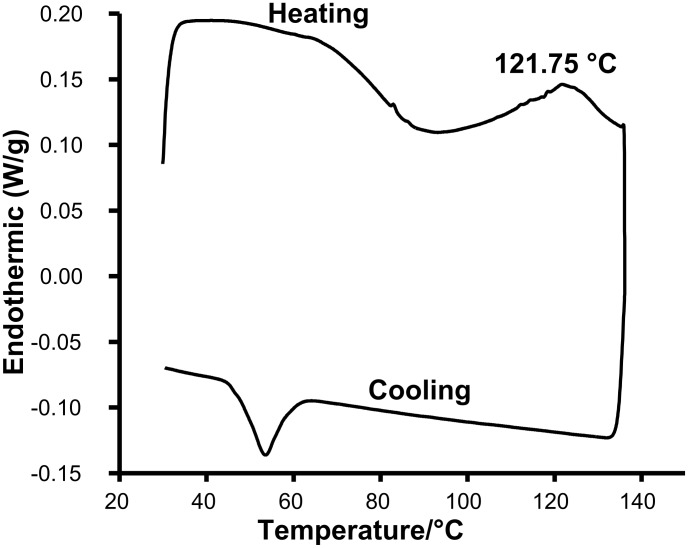
DSC of a 4.0 wt % 1,2-dichlorobenzene gel of DBAMC **6**.

To ascertain the thermoreversibility of the gel network, DSC was recorded on a selected gel sample derived from a ~4.0 wt % 1,2-dichlorobenzene solution of DBAMC **6** (Figure 2).

**Figure 3:**
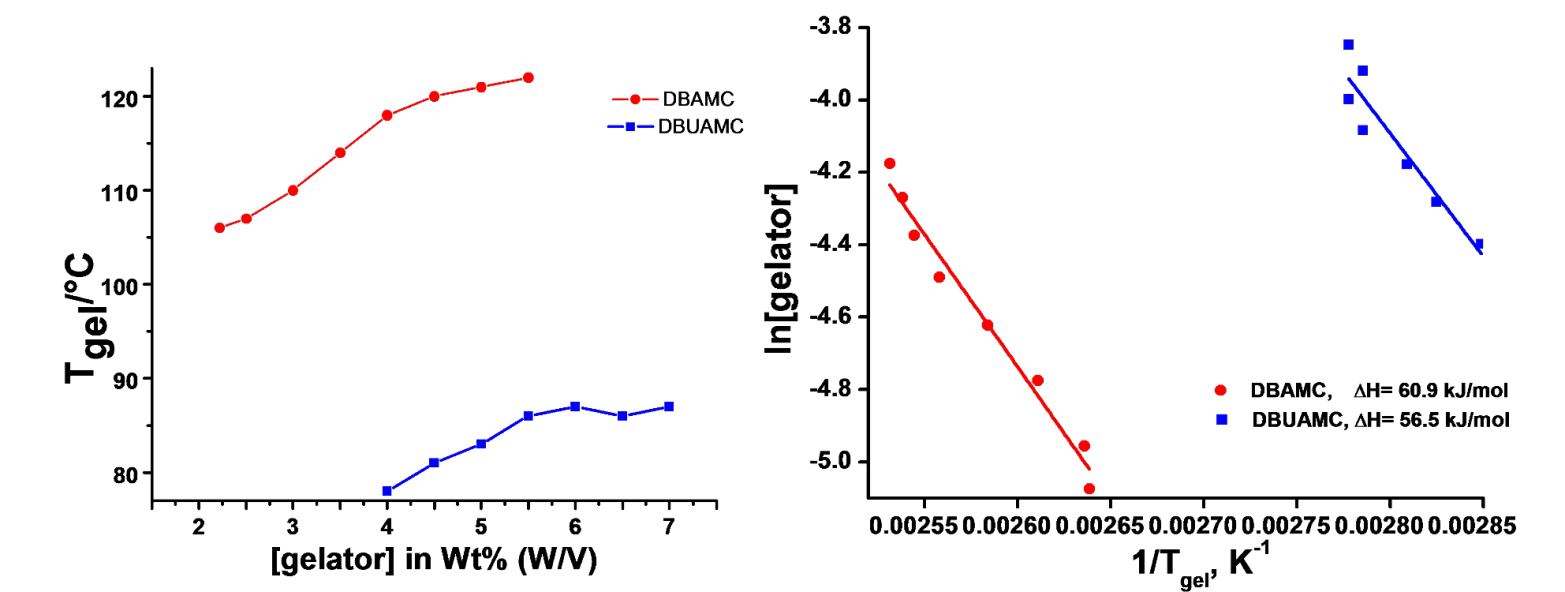
*Left* – *T*_gel_ vs [gelator] plot; *right* – semilog plot of mole fraction of the gelators against 1/*T*; 1,2-Dichlorobenzene and nitrobenzene gels were used for DBAMC **6** and DBUAMC **3**, respectively.

It is clear from the DSC data that the gelation was indeed thermoreversible. However, both the sol-gel and gel-sol transitions occur over a broad range of temperature making it difficult to assess the enthalpy change associated with this process. To get some idea about the enthalpy change associated with gel-sol, we carried out table top rheology [48] on some selected gels (Figure 3).

*T*_gel_ (gel-sol dissociation temperature) vs [gelator] plots on some selected gels displayed a steady increase of *T*_gel_ with the increase in [gelator] which indicated that, in the present cases, self-assembly in the gel state was driven by strong supramolecular interactions such as hydrogen bonding. Application of the Schroeder-van Laar equation (Equation 1) resulted in a linear semilog plot (Figure 3), when the mole fraction of the gelator at each concentration was plotted against 1/*T*_gel_ K^−1^.

[1]
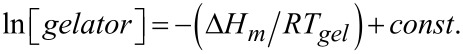


Where Δ*H*_m_ and *T*_gel_ are the enthalpy change and temperature associated with the gel-sol transition process, respectively and *R* is universal gas constant. Here it is considered that gel-sol transition is first order in nature on the assumption that the gel melts into an ideal solution wherein the exact amount of gel involved in the transition is known. The calculated Δ*H* value for DBAMC **6** is 60.9 kJ/mol and that of DBUAMC **3** is 56.5 kJ/mol, respectively which clearly indicates that 1,2-dichlorobenzene gel of DBAMC **6** is stronger than the nitrobenzene gel of DBUAMC **3**.

**Figure 4:**
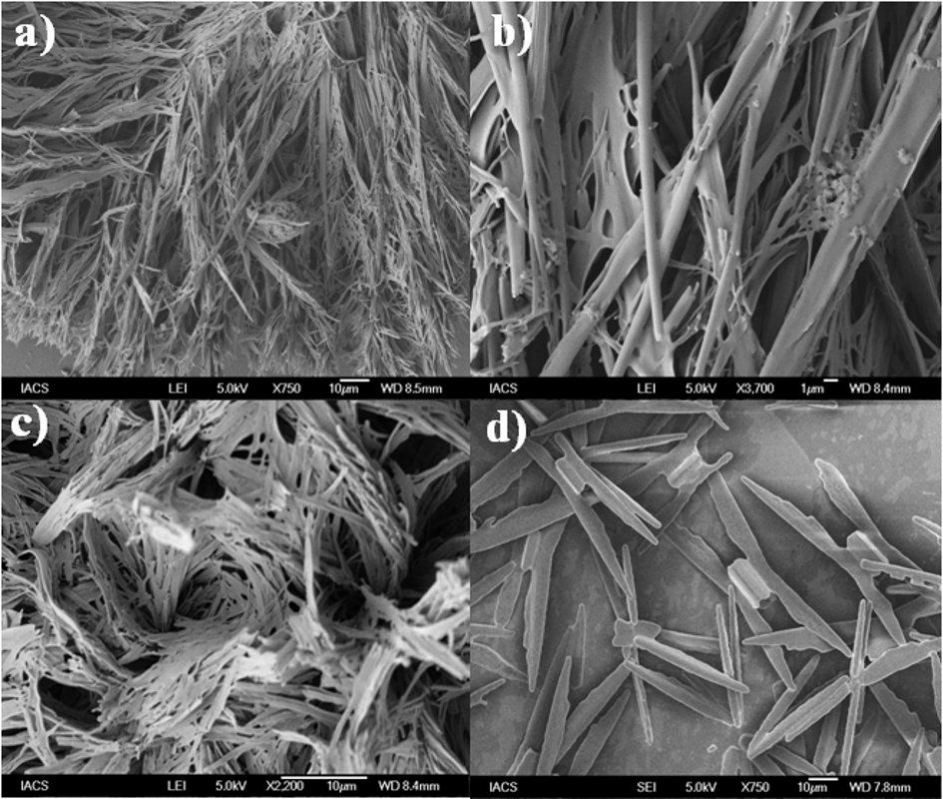
SEM micrographs of the xerogels. (a) & (b) 0.5 wt % 1,2-dichlorobenzene gel of DBAMC **6**; (c) 0.8 wt % chlorobenzene gel of DBAMC **6**; d) 0.5 wt % nitrobenzene gel of DBUAMC **3**.

To see the morphological features of the gel fibers, some selected xerogels were subjected to SEM (Figure 4). Highly entangled networks of fibers were seen in the chlorobenzene and 1,2-dichlorobenzene xerogels of DBAMC **6**, whereas relatively short plate like morphology was observed in the nitrobenzene xerogel of DBUAMC **3**. Understandably, the solvent molecules are immobilized in these networks to form gel.

To prove structure-property correlation in these gelators, we tried to crystallize as many salts as possible. However, our best efforts resulted in the crystallization of only three salts, DBUAMC **3**, DBAMC **6** and DCHADC **1**, which were examined by single crystal X-ray diffraction (Table 2).

**Table 2:** Crystallographic data.

Crystal parameters	DBUAMC 3	DBAMC 6	DCHADC 1

Empirical formula	C_18_H_33_NO_4_	C_24_H_31_NO_4_	C_34_H_62_N_2_O_4_
Formula weight	327.45	397.50	562.86
Crystal size/mm	0.46 × 0.38 × 0.28	0.24 × 0.19 × 0.12	0.28 × 0.16 × 0.12
Crystal system	Orthorhombic	Monoclinic	Monoclinic
Space group	*P*2_1_2_1_2_1_	*P*2_1_	*P*2_1_
*a* / Å	8.6977(9)	6.6454(3)	12.2424(15)
*b* / Å	12.5877(13)	17.9624(9)	17.278(2)
*c* / Å	18.8825(19)	9.3062(4)	16.7260(19)
α / °	90.00	90.00	90.00
β / °	90.00	98.981(4)	98.199(2)
γ / °	90.00	90.00	90.00
Volume / Å^3^	2067.3(4)	1097.24(9)	3501.8(7)
Z	4	2	4
F(000)	720	428	1248
μ MoKα / mm^−1^	0.073	0.081	0.068
Temperature / K	298(2)	100(2)	298(2)
*R*_int_	0.0368	0.0397	0.0453
Range of *h*, *k*, *l*	−10/10, −14/9, −17/22	−10/10, −7/7, −18/17	−12/13, −18/13, −17/16
θmin / max / °	1.94 /25.00	2.49/26.00	1.23 / 22.50
Reflections collected/unique/observed [I>2σ(I)]	8622 /3609 /3015	11570/4209/2685	11933/6369/5344
Data/restraints/parameters	3609/0/204	4209/1/266	6369/1/727
Goodness of fit on *F*^2^	1.090	0.923	1.219
Final R indices [I>2σ(I)]	R_1_ = 0.0724 wR_2_ = 0.2043	R_1_ = 0.0462 wR_2_ = 0.1039	R_1_ = 0.1042 wR_2_ = 0.2406
R indices (all data)	R_1_ = 0.0820 wR_2_ = 0.2230	R_1_ = 0.0845 wR_2_ = 0.1153	R_1_ = 0.1194 wR_2_ = 0.2529

**Figure 5:**
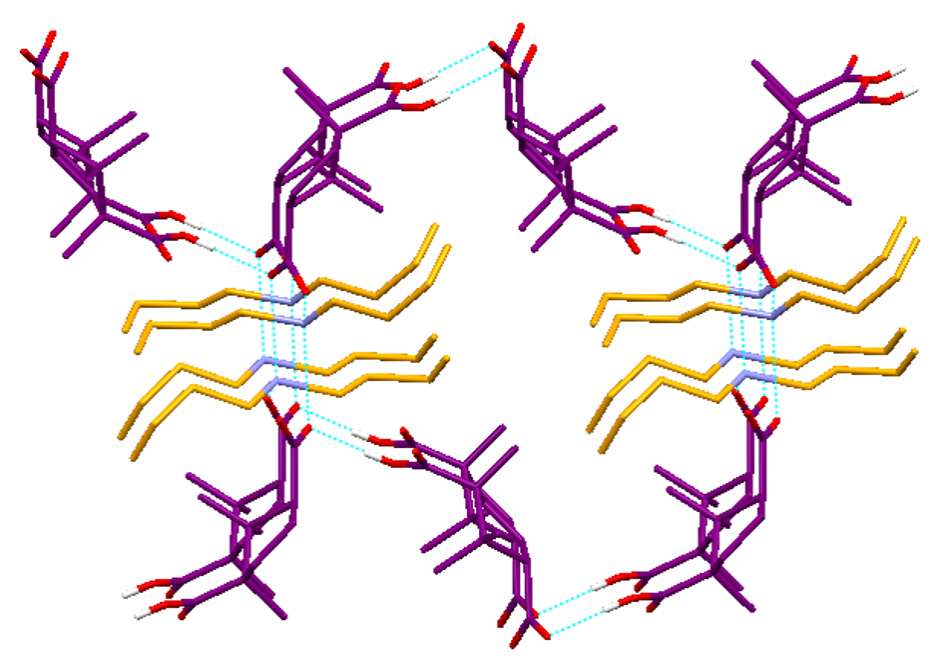
Crystal structure illustration of DBUAMC **3**; 3-D hydrogen bonded network; only one part of the disordered ammonium cation and hydrogen atom associated with carboxylic moiety are shown for clarity.

The crystal of DBUAMC **3** isolated from ethylene glycol/methanol mixture belongs to the orthorhombic space group *P*2_1_2_1_2_1_. The carboxylic acid moiety shows the C–O distances as 1.241(3)–1.272(3) and 1.197(4)–1.300(4) Å which is indicative of the presence of both COOH and COO^−^. FT-IR data also support this observation (1701 and 1631 cm^−1^). The presence of a secondary ammonium cation is also evident from the strong peak at 2960 cm^−1^ with multiple bands extending to 2411 cm^−1^. In the crystal structure, the butylammonium cation is disordered over two positions. The strongest hydrogen bonding donor, the charge assisted secondary ammonium cation, form hydrogen bonds with the strongest hydrogen bonding acceptor COO^−^; interestingly, the COO^−^ forms hydrogen bonding with two crystallographically equivalent dibutylammonium cations [N…O = 2.725(7)–3.040(6) Å]. On the other hand, the COOH moiety forms hydrogen bonding only with COO^−^ [O…O = 2.614(3) Å; 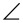 O–H…O = 176.9°]. Such hydrogen bonding interactions lead to the formation of a 3-D hydrogen bonded network (Figure 5).

**Figure 6:**
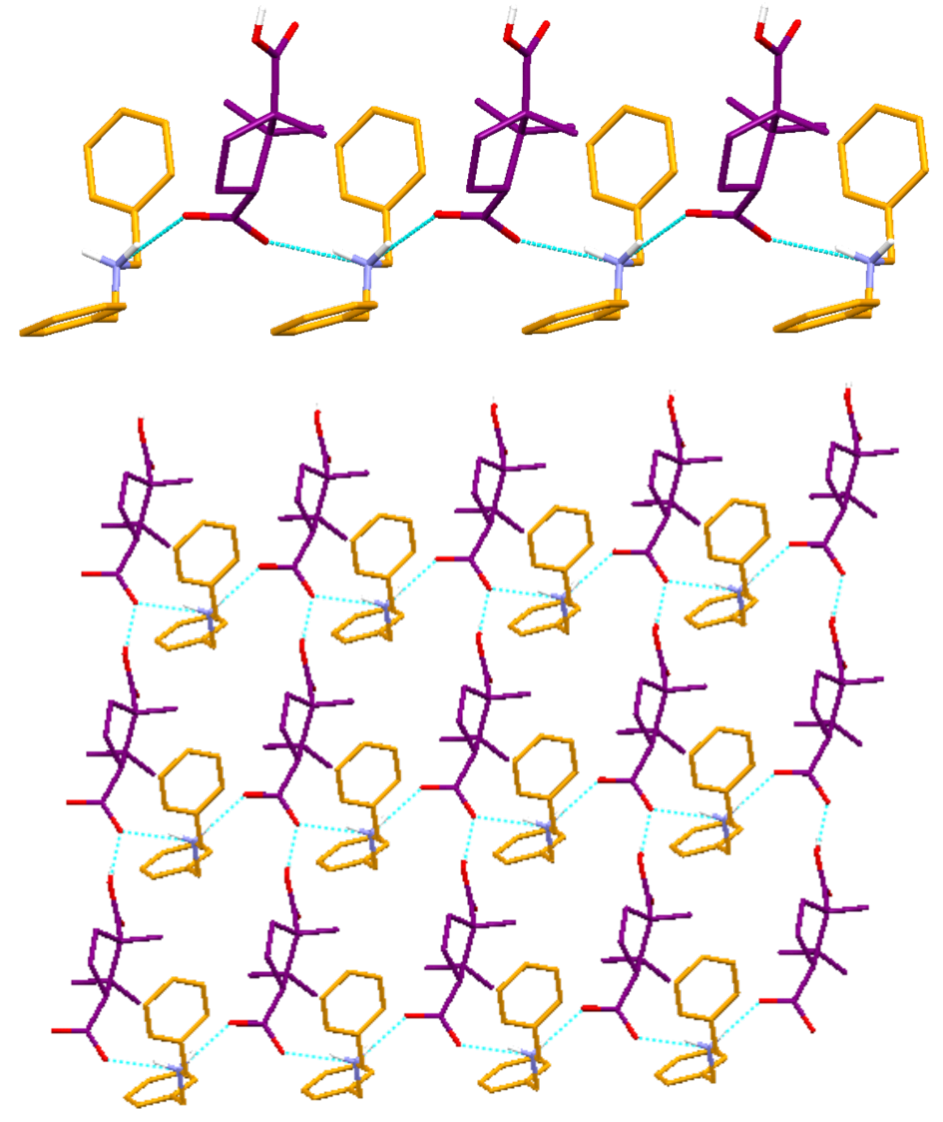
Crystal structure illustrations of DBAMC **6**; *top* – propagation 1-D network involving ammonium and carboxylate ions; *bottom* – overall 2-D hydrogen bonded network.

Crystals of DBAMC **6** suitable for single crystal X-ray diffraction study were grown from mesitylene. It crystallized in the non-centrosymmetric monoclinic space group *P*2_1_. The C–O distances of the carboxylic acid moieties are 1.237(2)–1.270(3) Å and 1.193(3)–1.309(3) Å indicating that only one COOH group is deprotonated. This is also evident in the FT-IR spectra of **6** wherein bands characteristic of COOH (1705 cm^−1^) and COO^−^ (1548 cm^−1^) were observed. A strong band at 2974 cm^−1^ with multiple bands extending to 2445 cm^−1^ also supports the existence of secondary ammonium cation. In the crystal structure, the strongest hydrogen bonding donor, the charge assisted secondary ammonium cation, and the acceptor (the carboxylate anion) are involved in hydrogen bonding [N…O = 2.711(2)–2.752(2) Å; 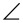 N^+^–H…O = 161.3–168.6°] resulting in 1-D hydrogen bonded network. The COOH group bridges such 1-D chains by O–H…O hydrogen bonding [O…O = 2.570(2) Å; 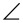 O–H…O = 161.38°] involving COOH and COO^−^ resulting into a overall 2-D hydrogen bonded sheet that runs along the c-axis. The 2-D sheets are further packed in a parallel fashion along the b-axis sustained by weak π-π stacking interactions (3.926 Å) involving the phenyl groups of the neighboring 2-D sheets (Figure 6).

**Figure 7:**
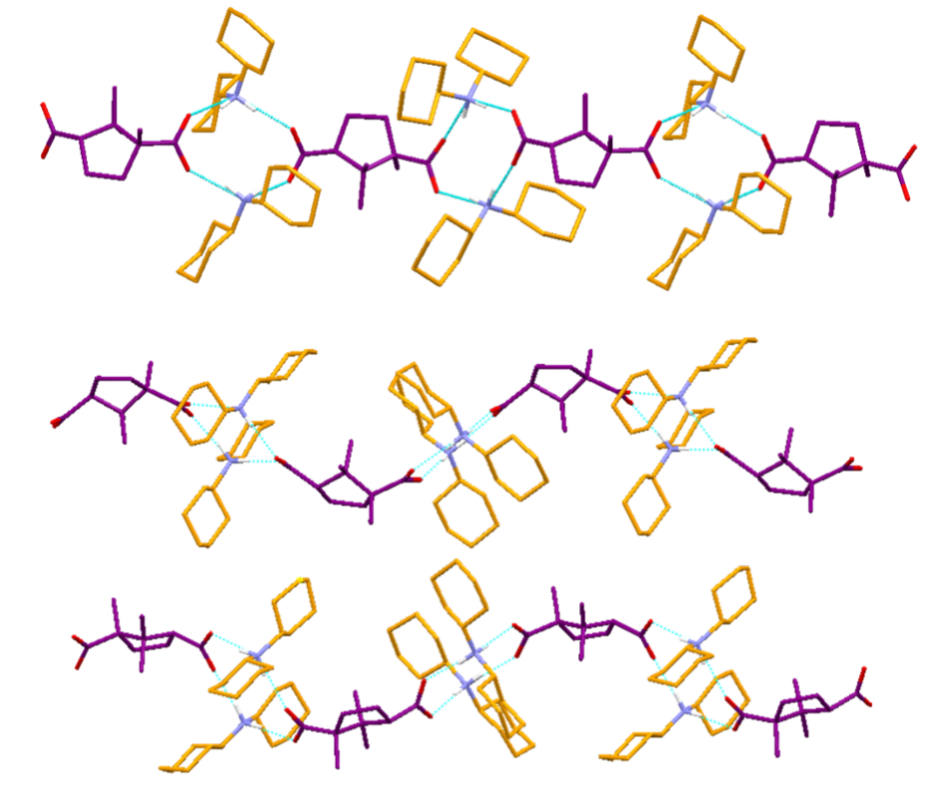
Illustration of crystal structure of DCHADC **1**; *top* – 1-D hydrogen bonded zigzag chain displaying SAD synthon; *bottom* – packing of such zigzag chains.

Crystals of DCHADC **1** was grown from m-xylene. It was crystallized in the non-centrosymmetric monoclinic space group *P*2_1_. The C–O distance of the carboxylic acid moieties are 1.226(10)–1.259(10)) Å and 1.226(10)–1.233(11) Å indicating that both the COOH groups are deprotonated which is consistent with the FT-IR data. The appearance of one band at 1622 cm^−1^ and absence of COOH band at 1699 cm^−1^ for the parent acid suggest that both the carboxylic acid groups are deprotonated. A strong band at 2928 cm^−1^ with multiple bands extending to 2362 cm^−1^ also supports the existence of secondary ammonium cation. In the crystal structure, the strongest hydrogen bonding donor, the charge assisted secondary ammonium cation, and the acceptor – the carboxylate anion – undergo hydrogen bonding [N…O = 2.653(9)–2.742(10) Å; 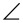 N^+^–H…O = 159.5–169.1°] resulting in 1-D zigzag hydrogen bonded network. Because of the bifunctionality of the camphorate moiety, this network propagates in one direction, resulting in 1-D zigzag networks, which are arranged in a parallel fashion in the crystal lattice (Figure 7).

Thus, it is clear that both salts **3** and **6** are1:1 acid:base salts and obviously do not possess SAD moieties, whereas salt **1**, which is a 1:2 acid:amine salt, does indeed have a SAD synthon. However, salts **3** and **6** were able to gel a few solvents, whilst salt **1** failed to gel any of the solvents studied herein. It may be recalled here that 2-D hydrogen bonded networks (such as in the salts **3** and **6**) have been shown to play a crucial role in gelation [3]. The failure of the salt **1**, displaying 1-D SAD synthon, to form gels once again points to the need for a better understanding of gel fiber and solvent interactions.

**Figure 8:**
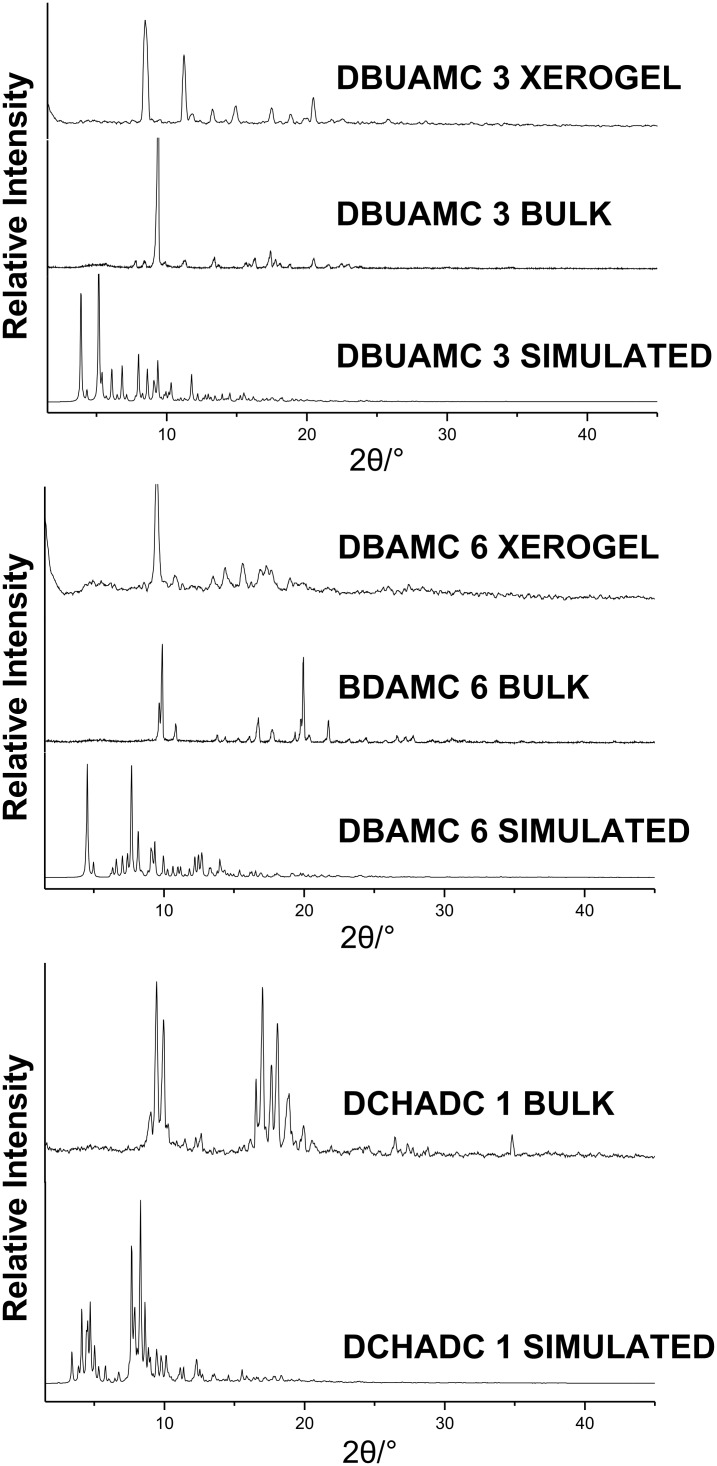
PXRD patterns of salts **3**, **6** and **1** under various conditions.

To see if these crystal structures of **3** and **6 (**as discussed above) truly represent the bulk solid as well as the xerogels, we undertook detailed PXRD studies. The comparison plot involving simulated, bulk and xerogel PXRDs for both the salts do not match which indicate the presence of other morphs in the bulk as well as in the corresponding xerogels. The single crystal structure of the salt **1** also appears to be unrepresentative of its bulk as evident from the PXRD comparison plots of the simulated and bulk solid (Figure 8).

## Conclusion

We have synthesized a series of secondary ammonium salts of (1*R,*3*S*)-(+)-camphoric acid following the rationale of supramolecular synthon in order to have an easy access to chiral gels. Out of seven salts prepared, four were 1:2 acid:amine salts, whereas the others were 1:1 salts. Two 1:1 salts, i.e., DBUAMC **3** and DBAMC **6** were found to be moderate gelling agents. The rest of the salts were either non-gelators or showed weak gelation abilities. Table top rheology data suggest that the 1,2-dichlorobenzene gel of DBAMC **6** is stronger than the nitrobenzene gel of DBUAMC **3**. Attempts to correlate the structure with gelling/non-gelling behavior based on various X-ray diffraction techniques was inconclusive as the PXRD patterns of the simulated, bulk and xerogel do not match in both the gelators. Moreover, salt **1** which displayed 1-D SAD synthon failed to gel any of the solvents studied herein indicating that many factors that might be crucial for gelation such as the nucleation of gel fiber, kinetics of gel fiber growth, their self-assembly to form SAFINs and their interactions with the solvent molecules etc. are needed for a deeper understanding. Although we were successful in achieving an easy access to few chiral gels following this supramolecular synthon approach, this study clearly indicates that some of the integrated parts associated with the gelation phenomena require to be better understood before a straightforward design strategy for synthesizing gelling agents can be formulated.

## Experimental

### Materials and physical measurements

All the reagents were obtained from various commercial sources (Sigma-Alrdrich, S. D. Fine Chemical,India etc.) and used as such without further purification. Solvents were of L. R. grade (Ranchem, Spectrochem, India etc.) and were used without further distillation. Melting points were determined by Veego programmable melting point apparatus, India. IR spectra were obtained on a FT-IR instrument (FTIR-8300, Shimadzu). The elemental compositions of the purified compounds were confirmed by elemental analysis (Perkin-Elmer Precisely, Series-II, CHNO/S Analyzer-2400). Scanning electron microscopy (SEM) was carried out with a JEOL, JMS-6700F, Field Emission Scanning Electro Microscope. Differential Scanning Calorimetry (DSC) was recorded with a Perkin-Elmer, Diamond DSC. Powder X-ray patterns were recorded on a Bruker AXS D8 Advance Powder (Cu Kα1 radiation, λ = 1.5406 Å) diffractometer.

#### General Synthetic Procedure

The salts were synthesized by reacting the acid and the corresponding amine in a 1:2 molar ratio (except for DBUAMC **3**, DBAMC **6** and DSBUAMC **7** where the stoichiometry of acid and amine were 1:1) in MeOH in a beaker. The resultant mixture was subjected to sonication for a few minutes to ensure the homogeneous mixing of the two components. The resulting mixture was then kept at room temperature from which a white solid was collected in near-quantitative yield after 1–2 days and then subjected to various physicochemical analyses and gelation test. All the salts were fully characterized by FT-IR and elemental analysis (except for DBUAMC **3** for which the elemental analysis data did match; however, other data such as FT-IR and single crystal X-ray indicated the formation of a 1:1 acid:amine salt).

#### *T*_gel_ Measurements

In a typical experiment, the salt was dissolved in the targeted solvent by heating. The solution was then allowed to cool to room temperature. Gel formation was confirmed by tube inversion. *T*_gel_ was measured by the dropping ball method; a glass ball weighing 242.0 mg was placed on a 0.5 mL gel in a test tube (10 × 100 mm). The tube was then immersed in an oil bath placed on a magnetic stirrer in order to ensure uniform heating. The temperature was noted when the ball touched the bottom of the tube.

#### Analytical data

DCHADC **1**: mp: 169–170 °C; FT-IR (cm^−1^): 2928, 2854, 2793, 2725, 2698, 2667, 2521, 2440, 2422, 2362, 2343, 2206, 2104, 1622, 1535, 1498, 1452, 1386, 1354, 1311, 1282, 1267, 1236, 1215, 1172, 1124, 1068, 1053, 1033, 1010, 977, 922, 889, 848, 798, 750, 597, 559, 499, 449, 412; Elemental analysis calculated for C_34_H_62_N_2_O_4_: C, 72.55; H, 11.10; N, 4.98; Found: C, 72.42; H, 11.15; N, 5.05.

DPADC **2**: mp: 157–158 °C ; FT-IR (cm^−1^): 2966, 2939, 2879, 2845, 2806, 2704, 2565, 2443, 1633, 1533, 1467, 1458, 1384, 1354, 1327, 1309, 1280, 1182, 1122, 1057, 916, 877, 798, 756, 690, 551, 532, 482, 434; Elemental analysis calculated for C_22_H_46_N_2_O_4_: C, 65.63; H, 11.52; N, 6.96; Found: C, 65.62; H, 11.36; N, 6.86.

DBUAMC **3:** mp: 167–168 °C ; FT-IR (cm^-1^): 2960, 2933, 2874, 2837, 2785, 2580, 2478, 2411, 1701, 1631, 1537, 1462, 1383, 1354, 1329, 1311, 1284, 1259, 1172, 1124, 1080, 1057, 993, 914, 792, 754, 736, 476.

DIBUADC **4**: mp: 156–158 °C ; FT-IR (cm^−1^): 2964, 2875, 2850, 2559, 2428, 2360, 2339, 1635, 1535, 1465, 1381, 1352, 1307, 1282, 1172, 1120, 1080, 1035, 993, 796,758, 682, 673, 476, 430; Elemental analysis calculated for C_26_H_54_N_2_O_4_: C, 68.08; H, 11.87; N, 6.11; Found: C,67.56; H,11.50; N, 5.77.

DHADC **5**: mp: 114–115 °C; FT-IR (cm^−1^): 2958, 2931, 2860, 2575, 2459, 2418, 2364, 2341, 1631, 1539, 1464, 1381, 1354, 1327, 1313, 1280, 1215, 1170, 1122, 1080, 1062, 916, 796, 759, 729, 694, 547, 476; Elemental analysis calculated for C_33_H_68_N_2_O_4_: C, 71.17; H, 12.31; N, 5.03; Found: C,71.62; H,11.84; N, 4.97.

DBAMC **6**: mp: 184 °C; FT-IR (cm^−1^): 3053, 3032, 2974, 2928, 2879, 2744, 2590, 2445, 1952, 1705, 1548, 1498, 1458, 1396, 1369, 1294, 1234, 1207, 1114, 1082, 1049, 1026, 983, 910, 881, 779, 742, 694, 484, 455. Elemental analysis calculated for C_24_H_31_NO_4_: C, 72.52; H, 7.86; N, 3.52; Found: C, 72.27; H, 7.86; N, 3.37.

DSBUAMC **7**: mp: 116–117 °C; FT-IR (cm^−1^): 2976, 2941, 2881, 2779, 2737, 2600, 2497, 2434, 1701, 1620, 1552, 1456, 1392, 1371, 1300, 1244, 1207, 1112, 1035,1008, 977, 792, 725, 547, 466, 435. Elemental analysis calculated for C_18_H_35_NO_4_: C, 65.62; H, 10.71; N, 4.25; Found: C, 65.62; H, 10.14; N, 4.01.

#### X-ray single crystal data

Data were collected using MoKα (λ = 0.7107 Å) radiation on a BRUKER APEX II diffractometer equipped with CCD area detector. Data collection, data reduction, structure solution/refinement were carried out using the software package of SMART APEX. All structures were solved by the direct method and refined in a routine manner. In most of the cases, non-hydrogen atoms were treated anisotropically. All the hydrogen atoms were geometrically fixed. CCDC (CCDC No. 782834–782836) contains the supplementary crystallographic data for this paper. These data can be obtained free of charge via http://www.ccdc.cam.ac.uk/conts/retrieving.html (or from the Cambridge Crystallographic Data Centre, 12 Union Road, Cambridge CB21EZ, UK; fax: (+44) 1223-336-033; or deposit@ccdc.cam.ac.uk).

## Supporting Information

File 1 Cif file of crystal structure of **DBAMC 6**.

File 2 Cif file of crystal structure of **DBUAMC 3**.

File 3 Cif file of crystal structure of **DCHADC 1**.
